# Stabilizing mutations increase secretion of functional soluble TCR-Ig fusion proteins

**DOI:** 10.1186/1472-6750-10-61

**Published:** 2010-08-24

**Authors:** Elin Lunde, Geir Åge Løset, Bjarne Bogen, Inger Sandlie

**Affiliations:** 1 Centre for Immune Regulation; 2 Department of Molecular Biosciences, University of Oslo, Oslo 0316, Norway; 3 Institute of Immunology, University of Oslo and Oslo University Hospital, Oslo 0027, Norway

## Abstract

**Background:**

Whereas T cell receptors (TCRs) detect peptide/major histocompatibility complexes (pMHCs) with exquisite specificity, there are challenges regarding their expression and use as soluble detection molecules due to molecular instability. We have investigated strategies for the production of TCR-immunoglobulin (Ig) fusion proteins. Two different TCRs that are characteristic of a mouse model for idiotype (Id) dependent immune regulation were engineered. They are structurally unrelated with different variable (V), diversity (D) and joining (J) segments, but each share one V gene segment, either V_α _or V_β_, with the well characterized murine TCR, 2C.

**Results:**

Several TCR-Ig formats were assessed. In one, the TCR V domains were fused to Ig constant (C) regions. In others, the complete extracellular part of the TCR was fused either to a complete Ig or an Ig Fc region. All molecules were initially poorly secreted from eukaryotic cells, but replacement of unfavourable amino acids in the V regions improved secretion, as did the introduction of a disulfide bridge between the TCR C domains and the removal of an unpaired cysteine. A screening strategy for selection of mutations that stabilize the actual fusion molecules was developed and used successfully. Molecules that included the complete heterodimeric TCR, with a stabilizing disulfide bridge, were correctly folded as they bound TCR-specific antibodies (Abs) and detected pMHC on cells after specific peptide loading.

**Conclusions:**

We show that fully functional TCR-Ig fusion proteins can be made in good yields following stabilizing engineering of TCR V and C region genes. This is important since TCR-Ig fusions will be important probes for the presence of specific pMHCs *in vitro *and *in vivo*. In the absence of further affinity maturation, the reagents will be very useful for the detection of kinetic stability of complexes of peptide and MHC.

## Background

Whereas the use of recombinant soluble peptide-MHC (pMHC) molecules for identification of specific T cells has increased dramatically over the last years [1-3], the reciprocal approach of using recombinant soluble TCRs (that is, lacking the transmembrane and intracellular part) for specific detection of peptide presentation and targeting to specific pMHC on cells has proven far more difficult.

A few pMHC specific antibodies have been described, but are often cross-reactive [4-10]. The limitation may be overcome by the use of combinatorial antibody technology as demonstrated for pMHC class I [11]. However, neither antibody libraries, nor the full range of specific, recombinant pMHCs required for panning in the selection step, are readily available.

TCRs have evolved to recognize pMHC. They are detection molecules with exquisite specificity, and exhibit, like antibodies, an enormous diversity. Soluble TCRs also offer unique opportunities for novel, highly specific therapeutic molecules. Different approaches have therefore been taken for production and testing of soluble TCRs, most of which have been derived from established T cell clones of known specificity [12].

Soluble TCRs have been produced as heterodimers of α/β chains [13-15], or as two variable (V) domains joined in single chain TCRs (scTCRs) of various formats [16-20]. In general however, the development of such molecules is hampered by difficulties associated with low stability causing low expression yields, aggregation of purified proteins and misfolding [21].

In order to increase stability, the TCR V regions have been optimized by amino acid replacements. Such replacements have been described that increase the surface hydrophilicity of a scTCR derived from the human RFL 3.8 TCR [22], or yeast surface display [23] as well as resistance to thermal denaturation [24] of a scTCR derived from the murine 2C TCR.

In some cases, heterodimeric α/β TCRs have been stabilized by a non-native disulfide bridge between the constant (C) domains [25,26].

The intrinsic affinity of a TCR for its pMHC is in the lower micromolar range [27]. While all TCRs on the surface of a T cell are identical, only a few copies of a particular pMHC are displayed on the surface of an antigen presenting cell. Multimerization to increase avidity has therefore been obtained by either indirect capture on beads [28], direct biotinylation and binding to streptavidin [17] or by expressing TCRs on larger particles such as phage [29], viral capsids [19], or cells [30-32].

TCRs have been fused to other soluble polypeptides, amongst Igs, which have a number of advantages as fusion partner since they are naturally secreted, stable molecules. TCR-Ig molecules should be secreted and acquire increased stability and binding avidity upon dimerization, and detection of binding to target cells should be facilitated utilizing the vast repertoire of available methods developed for detection of Ig. In that way, one might tap into the successful strategies developed for monoclonal antibodies, including widely used purification methodology. In addition, the Fc region of TCR-Ig fusion proteins may well provide the targeting TCRs with effector functions *in vivo*, such as prolonged half life and other FcRn mediated effects, as well as the ability to kill target cells by complement activation and binding to Fc receptors.

However, early attempts to fuse TCRs to Ig domains failed to produce secreted TCRs [33-35]. Later, soluble TCR fusion proteins in which both TCR α- and β-chains were fused to simple C_κ _domains [36,37] were found to be secreted and recognized by anti-TCR Abs. More recently, a scTCR consisting of V_α_-linker-V_β_-C_β _was fused to the human IgG_1 _constant region [18].

Two TCRs have previously been fused to complete IgG_1 _and used to stain pMHC on cell surfaces as well as intracellularly after exogenous specific peptide loading [38,39]. We found that such molecules were secreted at very low levels, and therefore explored how select mutations might increase expression. We focused on stabilizing mutations, as no reports exist as to how stabilizing mutations in the TCR V or C domains may affect TCR-Ig fusion molecule production and specificity. Here, we describe the generation of a panel of TCR-Ig fusion proteins based on two different TCRs (4B2A1 and 7A10B2) that are specific for an Ig light chain CDR3 Id peptide presented on an MHC class II molecule in a mouse model for autoimmunity and tumour immunology [40-42]. Three different fusion formats were investigated.

## Results

### Design of TCR-Ig fusion proteins

**Figure 1:**
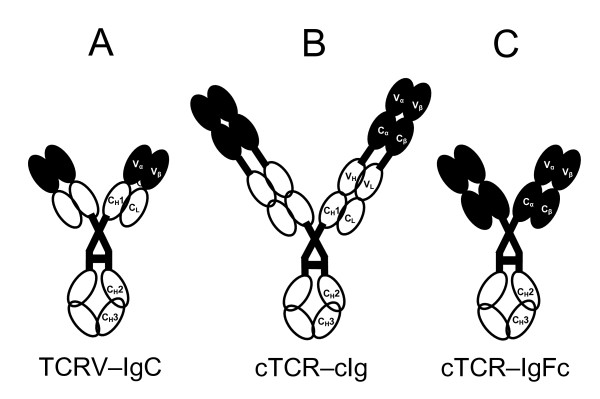
**TCR-Ig fusion protein formats**. In TCRV-IgC molecules, the Ig V regions are replaced by TCR V regions (**A**). cTCR-cIg molecules have the complete extracellular part of a TCR, C+V domains (cTCR) fused N terminally to a complete Ig (cIg) (**B**). In cTCR-IgFc molecules, the extracellular part of the TCR is fused to an Ig Fc region. TCR domains are filled and Ig domains are open circles (**C**). Notably, both the α and the β portions were tested as fusions to both the heavy and the light Ig chains in the TCRV-IgC (A) and cTCR-cIg (B) versions.

Two different TCRs were fused to Ig, namely 4B2A1 and 7A10B2. Both recognize amino acid 91-101 from the λ2^315 ^light chain of myeloma protein M315 presented on I-E^d ^MHC class II molecules [43]. The TCR-Ig proteins were made in three different fusion formats (Figure 1). In a first format, the TCR V regions were fused to IgG C regions (TCRV-IgC). In two other formats, both V and C TCR domains were included. Either, the extracellular domains of the TCRs were fused to a complete IgG (cTCR-cIg) or to the IgG hinge and Fc region (cTCR-IgFc). Thus, the term "cTCR" refers to the complete extracellular part of the TCR, including V and C regions, whereas "cIg" refers to complete Ig.

### The fusion proteins are secreted from HEK 293E cells

**Figure 2:**
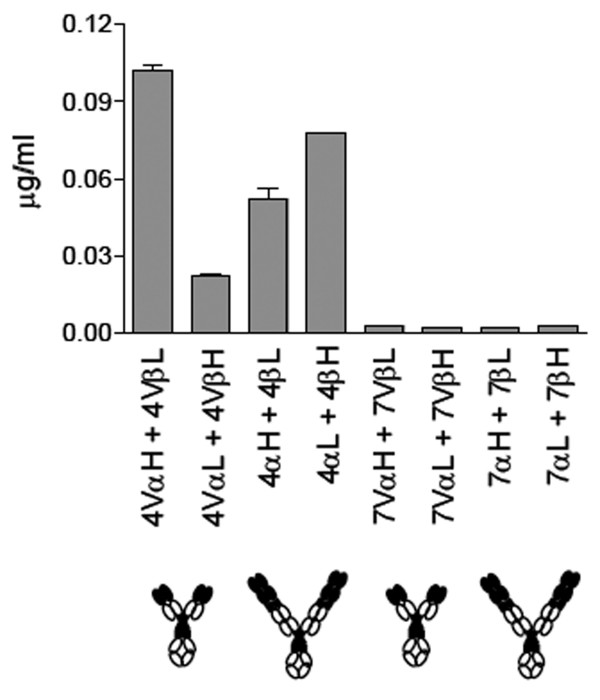
**Secretion of fusion proteins**. The four molecules to the left are derived from 4B2A1 (abbreviated 4), the four to the right from 7A10B2 (abbreviated 7). The TCR V_α _domain was fused to the heavy chain C region and the TCR V_β _domain on the light chain C region (V_α_H + V_β_L) or vice versa in the TCRV-IgC format. The complete TCR α chain was fused to the complete heavy chain and the TCR β chain fused to the light chain (_α_H + _β_L) or vice versa in the cTCR-cIg format. TCR domains are filled, Ig domains are open circles. Cell supernatant was tested in triplicates. Error bars: Standard deviation (SD). One representative experiment out of three is shown.

Genes encoding either TCRV-IgC or cTCR-cIg were assembled on vectors designed for Ig production in mammalian cells as described in Materials and Methods. For each TCR, both α and β chains were tested for fusion to both heavy and light Ig chains, a total of eight constructs. To investigate fusion protein secretion, HEK 293E cells were transiently transfected with the genes, and cell supernatants examined in ELISA specific for the Ig portion of the fusions after two days. Low secretion levels were observed in all cases (Figure 2). Fusion proteins based on the 4B2A1 receptor were secreted at higher levels than those based on 7A10B2. The fusion protein secreted at highest levels (~100 ng/ml) was the 4B2A1-based TCRV-IgC construct with the TCR V_α _domain fused to the heavy chain C region and the TCR V_β _domain fused to the light chain C region (V_α_H+V_β_L).

### Stability engineering of V regions improves secretion

To stabilize the TCR V domains and thus increase secretion levels, amino acids were replaced by site directed *in vitro *mutagenesis. A large number of mutants were generated and those that increased secretion levels in the actual TCRV-IgC format selected. Targeted positions have been described [22-24], and the substitutions tested were: S82R in 4B2A1 V_α_; G17E, H47Y, and L80S in 4B2A1 V_β_; L43P and W82R in 7A10B2 V_α_; as well as Q17E in 7A10B2 V_β_.

The mutations chosen have been identified in the context of the 2C TCR, which contains the V_β_8.2 and V_α_3.1 segments. While 4B2A1 shares the V_β_8.2 segment, 7A10B2 shares V_α_3.1 with 2C. Thus, when the V(D)J encoded complete V domain sequences are considered, 7B2A1 exhibits high homology to 2C V_α _(86.8% identity and 88.6% similarity), while 4B2A1 exhibits high homology to 2C V_β _(89.5% identity and 92.1% similarity).

The mutagenesis reactions were performed such that each gene acquired one or several alterations. All V_α_H variants were tested in combination with all V_β_L variants, and the best pair regarding secretion levels from transiently transfected cells identified for each receptor as described in Materials and methods_. _Analyses were done on the constructs that were secreted at the highest level in the previous section, namely TCRV-IgC with V_α_H+V_β_L.

**Figure 3:**
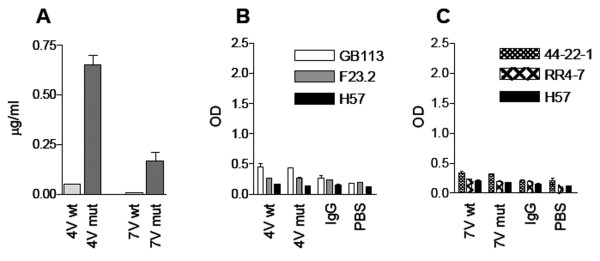
**Effect of V region mutations on secretion and folding of TCRV-IgC molecules of the (V**_**α**_**H+V**_**β**_**L) format**. Secretion of wild type (wt) and mutant 4B2A1- and 7A10B7-derived fusion proteins (4V mut and 7V mut) after transfection of HEK 293E cells. One representative experiment out of five is shown (**A**). Recognition by anti-TCR Abs (**B**). Wt and mutant 4B2A1 derived Ig-fusions were tested in ELISA using two Abs that recognize the 4B2A1 receptor (GB113 and F23.2) and H57, which is C region specific. One representative experiment out of three is shown (**B **and **C**). Wt and mutant 7A10B2 derived Ig-fusions were tested in ELISA using two Abs that recognize the 7A10B2 receptor (44-22-1 and RR4-7) and H57. All molecules were analyzed at the same concentration of 0.012 μg/ml. One representative experiment out of three is shown (**C**). Cell supernatant was tested in triplicates. Error bars: SD

For 4B2A1, the highest protein production was obtained when the wt α-chain was co-expressed with a β-chain that had acquired three mutations, namely G17E, H47Y, and L80S. As shown in Figure 3A, the mutant fusion protein, denoted 4 Vmut, was secreted thirteen times better than the wt. For 7A10B2, the selected combination was L43P and W82R in V_α _together with wt V_β_. The protein is denoted 7 Vmut, and production was increased seventeen fold (Figure 3A). Thus, amino acid replacements selected to improve the thermodynamic properties of 2C, also improved secretion of 4B2A1 as well as 7A10B2.

Folding was assessed in ELISA with a panel of anti-TCR mAbs as coat (Table 1). GB113, which is specific for 4B2A1 [44], and F23.2 [45], for which binding is conformation dependent and specific for the V_β_8.2 segment, were used for 4B2A1 [46]. 44-22-1 [47] and RR4-7 [48], both of which recognize the V_β_6 segment, were used for 7A10B2. The two 4B2A1 specific reagents do not recognize 7A10B2 and vice versa. In addition, both receptors were tested for binding to an anti-C_β _antibody, H57, which does not recognize this format which lacks C_β _(TCRV-IgC). Importantly, none of the TCRV-IgC molecules were recognized by the anti-TCR Abs (Figure 3B and 3C). Thus, even though the introduced mutations increased secretion, correct folding was not achieved. We therefore turned to other TCR fusion formats.

**Table 1:** TCR-specific Abs.

Ab clone	Specificity	T cell clone	References
F23.2	Murine TRBV13-2	4B2A1	[45,46]
H57-597	Murine C_β_	4B2A1 + 7A10B2	[64]
GB113	TCR 4B2A1	4B2A1	[44]
44-22-1	Murine TRBV19	7A10B2	[47,63]
RR4-7	Murine TRBV19	7A10B2	[48]

### Stability engineering of C regions improves folding and secretion

Comparing the cTCR-cIg molecules (Figure 1B) for secretion from HEK293E cells (Figure 2), both 4B2A1 derived molecule were secreted at medium levels. Molecules with complete α chain fused to the light chain and complete β chain fused to the heavy chain (αL + βH) showed the best secretion.

**Figure 4:**
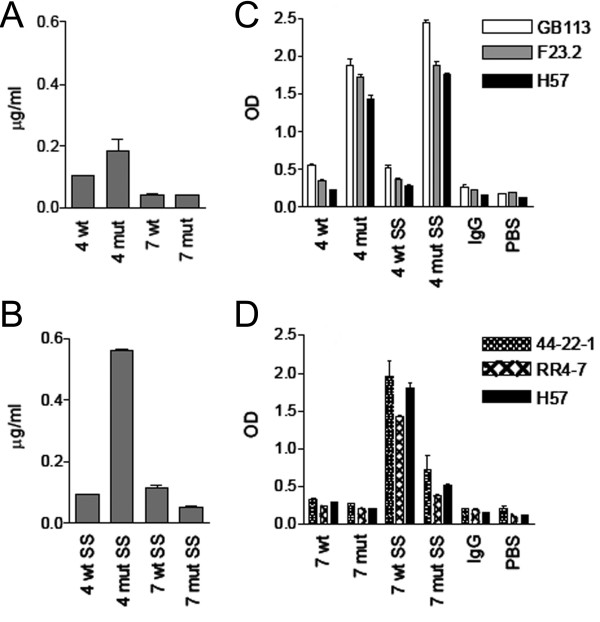
**Effect of V and C region mutations on secretion and folding of cTCR-cIg molecules produced in HEK 293E cells**. Secretion of molecules with or without V region mutations (4 mut and 7 mut). One representative experiment out of three is shown (**A**). Secretion of molecules with or without V region mutations and C regions with an introduced disulfide bridge (denoted SS and mutSS). One representative experiment out of three is shown (**B**). Recognition by anti-TCR Abs. **C**. The 4B2A1 derived fusions were tested for binding to 4B2A1 specific mAbs. One representative experiment out of two is shown (**C **and **D**). The 7A10B2 derived fusions were tested for binding to the 7A10B2 specific mAbs. H57 is C_β _region specific. One representative experiment out of two is shown (**D**). Cell supernatant was tested in triplicates. Error bars: SD

The selected V region mutations were then introduced in molecules of the cTCR-cIg format derived from both 4B2A1 and 7A10B2 (denoted 4 mut and 7 mut), the molecules produced in HEK293E cells and secretion levels measured by ELISA as above. For 4 mut (4B2A1 cTCR-cIg), secretion doubled, whereas no change was observed for 7 mut (Figure 4A). Subsequently, the TCR C regions were modified by the introduction of a disulfide bridge between the two C domains (mTRAC T48C and mTRBC S57C) and by replacing an unpaired cysteine in the β-chain with alanine, as previously described [29].

This was done in wt as well as in V region mutated molecules denoted 4wtSS, 4mutSS a.s.o. For 4wtSS, the introduced disulfide bridge did not alter the secretion level. However, when C region mutations were combined with stabilizing V region mutations (4mutSS), protein production was improved five-fold (Figure 4B). For 7wtSS, secretion increased two fold. There was no additive effect of V and C region mutations for the 7A10B2 derived molecule.

Folding was assessed in ELISA using anti-TCR Abs. Both 4 mut and 4 mutSS were well recognized by all relevant antibodies (Figure 4C). For the corresponding 7A10B2 derived receptor (Figure 4D), the V region mutations alone did not improve recognition. Introduction of the disulfide bridge, however, did. The TCR Cβ region specific mAb, H57, bound both 4B2A1 and 7A10B2 derived molecules with the same efficiency as the V region specific mAbs.

Two fusion molecules that were well recognized by TCR-specific antibodies, namely 4 mutSS and 7 wtSS, were secreted at 0.6 and 0.1 μg/ml, respectively (Figure 4B). Using the NS0 myeloma cell line, the production rate was lower, and the supernatants of stably transfected cells regularly contained approximately 1/10 the amount of fusion protein as that of the HEK 293E supernatants (data not shown). In comparison, IgGs can be obtained from HEK293E cells at 10 μg/ml 2 days after transient transfection. This prompted us to investigate whether production of TCR-Igs could be increased in insect cells.

### The fusion proteins are secreted from insect cells

**Figure 5:**
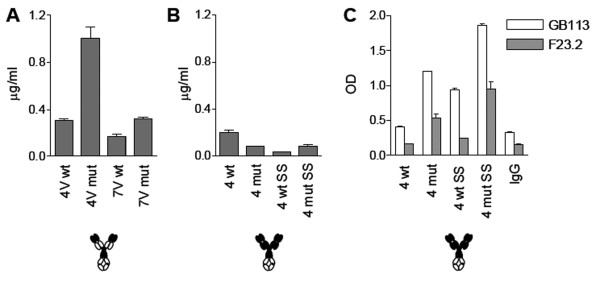
**Effect of V and C region mutations on secretion and folding of TCRV-IgC and cTCR-IgFc molecules produced in insect cells**. Secretion of 4B2A1 derived TCRV-IgC fusion proteins with or without V region mutations. One representative experiment out of three is shown (**A**). Secretion of 4B2A1 derived cTCR-IgFc fusion proteins with or without V and/or C region mutations. One representative experiment out of three is shown (**B**). Recognition by anti-TCR 4B2A1 specific Abs. One representative experiment out of two is shown. TCR domains are filled, and Ig domains open circles (**C**). Insect cell supernatant after three rounds of amplifications was tested in triplicates. Error bars: SD.

Initially, we compared mammalian and insect cell production of the TCRV-IgC format. The complete fusion genes were transferred from pLNOH2 and pLNOκ to pAc-κ-Fc, which is designed for Ig production using baculovira and insect cell infection. The Fc encoding gene originally in the vector was deleted. Both 4B2A1 and 7A10B2 receptors were tested. We found that the secretion levels doubled in Sf9 insect cells compared to HEK 293E cells (Figure 5A). The effect of V region mutations was less prominent, however, and again, the molecules were not recognized by anti-TCR Abs (results not shown).

We then introduced the cTCR encoding genes upstream of the Fc region encoded in the pAc-κ-Fc vector. The 4B2A1 receptor, with or without V region mutations as well as with or without C region mutations, was tested, and a total of four different molecules were included in the experiment. In all, the 4B2A1 α-chain was fused to the Fc region, whereas the β-chain was not part of a fusion. In general, the secretion levels were decreased relative to those obtained in mammalian cells (Figure 5B). As for the corresponding proteins produced in HEK 293E cells, both V and C region mutations increased anti-TCR Ab recognition, and the effects were additive (Figure 5C). In conclusion, correctly folded fusion molecules were obtained in insect cells after introduction of V and C region mutations, and the production levels were the same as that obtained in mammalian cells 2 days after transient transfection.

### The stability engineered fusion proteins bind specifically to pMHC on cells

**Figure 6:**
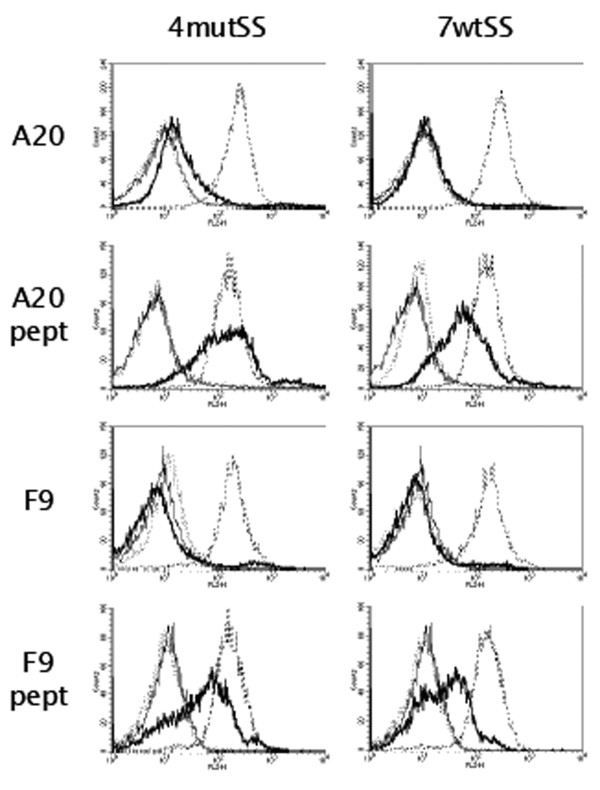
**Binding of TCR-Ig to specific pMHC expressing cells**. A20 cells (top panel), A20 cells loaded with synthetic peptide (A20 pept, second panel), F9 cells (third panel), and F9 cells loaded with synthetic peptide (F9 pept, forth panel) were stained with the 4B2A1-based purified cTCR-cIg protein (4mutSS, 100 μg/ml, all left panels) and the 7A10B2-based cTCR-cIg protein (7wtSS, 17 μg/ml, all right panels). Thin line, PBS control; dotted line, NIP-specific Ab (isotype matched); thick line: cTRC-cIg fusion protein; broken line, I-E-specific Ab (isotype-matched). In each case, 10^6 ^cells were stained after pulsing twice with 50 μM peptide, both 18 and 2 hours before the experiment was carried out. One representative experiment out of three is shown.

The molecules 4 Vmut and 7 Vwt (TCRV-IgC) as well as 4 mutSS and 7 wtSS (cTCR-cIg) were tested for binding to A20 cells in flow cytometry. A20 is a B cell lymphoma line that expresses the I-E^d ^molecule. We loaded the cells with synthetic λ2^315 ^peptide and found that 4 mutSS and 7 wtSS bound (Figure 6), whereas 4 Vmut and 7 Vmut, neither of which bound anti-TCR Abs, did not (data not shown). For both 4 mutSS and 7 wtSS, binding was clearly peptide-specific, as pMHC was detected only after addition of peptide. The concentration of fusion protein used, was 100 μg/ml for 4 mutSS and 17 μg/ml for 7 wtSS, respectively. When reducing the concentration of 4 mutSS to 17 μg/ml, the staining intensity was comparable to that of 7A10B2 (data not shown). Binding was also compared to that of a recombinant isotype-matched mAb with I-E-specificity [49]. For both TCR fusion proteins, staining intensities were comparable to those of the mAb (Figure 6). F9 cells are A20 transfected with λ2^315^. We have previously demonstrated that F9 presents the λ2^315 ^peptide on I-E^d ^and induces proliferation of specific T cells [50].

We tested whether the four molecules could distinguish between A20 and F9. Binding was not observed to either without addition of exogenous peptide. This shows that the reagent requires further affinity maturation to detect physiological concentrations of agonist peptide.

## Discussion

Fusion to Ig might facilitate expression, purification, as well as recognition of soluble TCRs bound to target pMHC. Furthermore, fusion of one TCR onto each "arm" of the Ig molecule ensures TCR dimer formation in the final TCR-Ig molecule, and consequently increased pMHC binding strength due to avidity effects.

Recent advances in TCR engineering include the identification of stabilizing mutations in both V- and C regions. We therefore investigated how such engineering affected production and ligand binding properties of two well characterized TCRs fused to Ig.

The best domain pairing when the TCR V regions substitute the Ab V regions in the TCRV-IgC format, V_β_C_H_/V_α_C_L _or V_α_C_H_/V_β_C_L, _may not easily be predicted, since the TCR C_α _domain deviates from the Ig fold and has no clear homology with any of the Ig C domains [51]. We found V_α_C_H_/V_β_C_L _to be secreted at the highest level when the TCRV-IgC format was investigated for production yield.

**Figure 7:**
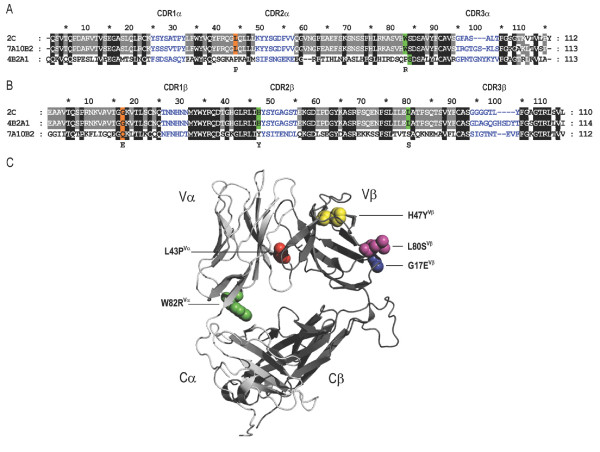
**Structural alignment of the V domains from the 4B2A1, 7B2A1 and 2C TCRs**. Alignments of the Vα (**A**) and Vβ (**B**) were made with MUSCLE [67] and annotated according to Hare *et al*. [68]. Amino acid positions mutated are indicated by colour code according to the source, orange[23] and green [24]), and the resulting residues are indicated at each position. 4B2A1 was originally misaligned regarding α chain residue 82. Ribbon representation of the 2C crystal structure (PDB ID: :1TCR) with the positions of the five mutations selected in the 4Vmut and 7Vmut variants, as given by the alignments in A and B (shown as spheres). The figure was prepared using PyMol (**C**).

The TCR V regions were then optimized by amino acid replacements introduced into this TCRV-IgC fusion format, as shown in Figure 7A for V_α _and 7B for V_β_. TCR 2C and 7A10B2 share V_α _segment, and the two V_α _replacements that were beneficiary for 7A10B2 were first characterized in the context of a scTCR version of the 2C TCR. For 4B2A1V_α_, the wt sequence was selected, which already contains one of these.

4B2A1 and 2C share V_β _segment, and all three V_β _replacements previously selected in the context of 2C were also selected for 4B2A1. 7A10B2 utilizes another V_β _gene segment, and again, this already contains the beneficiary amino acids.

The effect of the V domain mutations may be studied at the atomic level in the crystal structure of a mutated scTCR version of the 2C TCR (2C-T7) [52] and was recently thoroughly analyzed by Richman *et al*. [53]. Figure 7C shows a ribbon representation of 2C TCR with the five mutated positions highlighted. The V_β _segment was clearly well stabilized by the mutations, while the V_α _segment was not. This emphasizes the need for further stability engineering. Such engineering by yeast surface display has been reported for several TCRs of both mouse and human origin [24,54]. The widely versatile phage display technology could develop the engineering beyond its present state by selecting for features such as resistance to aggregation after acid or heat exposure as described for antibodies [55,56]. Following initial selection, pools of V domains may be transferred from the displayed scTCR format into a TCR-Ig format by the method described here.

Folding was analyzed employing a panel of anti-TCR Abs with V region specificity, and the results strongly suggested that all detected TCRs were correctly folded. In addition, the C region specific Ab H57 bound exactly the same molecules as the Abs with V region specificity. Thus, correct folding of the C domain could be used as an indicator of correct V domain folding, while erroneous V domain folding, when detected, was never a local event, but also affected the C domain. Secretion levels from producing cells were not a good indicator of correct folding, however. The TCRV-IgC format with TCR V regions and antibody C regions were well secreted, but not properly folded and this was true for both 4B2A1 and 7A10B2. The finding is not dependent on the nature of the eukaryotic production system, as the same observations were made whether the molecules were secreted from mammalian cells or from insect cells.

Native-like fold was readily reached upon reconstitution of the TCR V-C interphase for 4B2A1. This underscores the importance of this interphase whenever C domains are present in TCRs produced in eukaryotic cells. This also presented the opportunity for introduction of a disulfide bridge linking C_α _and C_β_. The corresponding bridge was first introduced with a positive effect on stability of human TCRs produced in *E. coli *[25]. Here we demonstrate that the bridge improved secretion of correctly folded cTCR-cIg molecules two (7A10B2) and five (4B2A1) fold, respectively, for murine TCRs. The V-C domain interphase analysis by Richman *et al*. [53] points to important differences in solvent exposure of the V domains in Abs and TCRs. In the native 2C TCR, V_α_W82 is burried in the V-C interphase, but is exchanged with a hydrophilic residue in the exposed scTCR version (W82R). This hydrophilic substitution also appeared beneficial in 7Amut selected in the TCRV-IgC format which has the V-C interphase of an Ab. In the cTCR-cIg format, however, the selected W82R mutation appeared counterproductive.

The fusion proteins were produced in different eukaryotic cells that secrete large proteins in a functional form, with disulfide bridges and glycosylation. HEK 293E cells were then chosen for large scale production and functional testing. The vectors used contain the oriP sequence that supports increased protein production in HEK 293E which express EBV nuclear antigen 1. Given frequent changes of growth medium, HEK 293E cells will continue to produce protein for 2 weeks [57]. During this time, the functional TCR-Ig fusion molecules could be obtained at 50 μg from an initial culture volume of 5 ml.

Specific binding to pMHC on cells was verified by flow cytometry after addition of the specific λ2^315 ^peptide. This underscores that the reagents produced are correctly folded and retained specificity, which was the major goal in this study. The reagents, like soluble TCR tetramers, will be very useful for the detection of kinetic stability of complexes of peptide and MHC [58]. Importantly, a difference in peptide:MHC stability was recently found to be related to autoimmune disease susceptibility [59].

B lymphoma cells that had been transfected with the λ2^315 ^gene were not detected. We have previously found that this particular transfectant stimulates cloned [50,60] or TCR-transgenic versions [61] of λ2^315^-specific T cells. A likely explanation is that the T cell based read-out, with aggregation of pMHCII/TCR in immunological synapses, is rather more sensitive than binding of soluble TCRs as detected in flow cytometry. In addition, coreceptors and costimulatory molecules on T cells do indeed contribute to the former - but not the latter - assay. In previous *in vitro *experiments, we found that soluble 4B2A1 TCRs displayed polyvalently on phage at 3-5 copies per particle bound A20 loaded with the same specific λ2^315 ^derived peptide as that used here, but not F9 [29]. Thus, increasing avidity beyond the dimeric Ig was not sufficient to detect physiological concentrations of pMHC. It is well known that the intrinsic affinity of a TCR for its cognate pMHC is most often in the lower micromolar range. The affinities of Fabs are also low in the primary humoral response. Ab binding to antigen occurs due to polymerization to pentamers (in reality 10 binding sites) in IgM in the primary response or affinity maturation in the secondary response. In the case of antigen presentation, the specific pMHC levels are so low that avidity is not going to operate. Thus, affinity maturation will be necessary to increase the sensitivity of the soluble TCR-Ig fusions to make them useful probes for physiological presentation of pMHC *in vitro *and *in vivo*.

## Conclusions

Manufacturing of stable recombinant Ig molecules is well established in a number of systems, and a large panel of reagents for detection of Ig exists. The extracellular parts of TCRs on the other hand, are difficult to produce and handle as recombinant soluble molecules, due to low intrinsic stability. Recent advances in TCR engineering include the identification of stabilizing mutations in both V- and C regions. We therefore investigated how such engineering affected production and ligand binding properties of two well characterized TCRs fused to Ig. Without engineering, the molecules were secreted at very low levels from different eukaryotic cells. However, improving the thermodynamic properties by TCR V region mutagenesis and the introduction of a disulfide bridge between the TCR C domains greatly improved yields. Most significantly, the engineered molecules bound specifically to pMHC on cells. The reagents will be very useful for the detection of kinetic stability of complexes of peptide and MHC.

## Methods

### Cells and antibodies

The 4B2A1 and 7A10B2 T cell clones have previously been described [43,62]. Both recognize amino acids 91-101 from the λ2 light chain of Ab produced by the MOPC315 plasmacytoma (λ2^315^) when presented on the MHC class II molecule I-E^d^.

The official IMGT (http://imgt.cines.fr/) gene segment nomenclature is used throughout. Thus, the following TCR gene segment re-designation of the murine T cell clones 4B2A1 [V_α_1, J_α_19/V_β_8.2, D_β_, J_β_1.2] and 7A10B2 [V_α_3, J_α_1/V_β_6, D_β_, J_β_1.1] is [TRAV7D-3*01, TRAJ40*01/TRBV13-2*01, TRBD1*01, TRBJ1-2*01] and [TRAV9-3*01, TRAJ58*01/TRBV19*01, TRBD1*01, TRBJ1-1*01], respectively.

Sf9, A20 and HEK 293E cells were from ATCC (Manassas, VA). F9 is A20 transfected with the λ2^315 ^light chain gene [50]. IgG-specific Abs used in ELISA where from Sigma-Aldrich (St. Louis, MO): two anti-human IgG_3 _(h IgG_3_) mAbs (HP6047 and HP6050), the latter being biotinylated, goat anti-hIgG Ab (I2136), as well as HRP-conjugated goat anti-hIgG Ab (A9544). TCR-specific mAbs were GB113 [44] (clonotype-specific for 4B2A1), F23.2 [45,46] (recognizes TRBV13-2), 44-22-1 [47,63] (recognizes TRBV19), RR4-7 [48] (recognizes TRBV19), and H57 [64] (recognizes murine C_β_). F23.2, H57, 44-22-1 were kind gifts from Dr. Uwe D. Staerz (Department of Medicine, National Jewish Medical and Research Center, Denver, USA), Dr. Ralph T. Kubo (Cytel Corporation, San Diego, USA), and Dr. Hans Hengartner (Institute for Experimental Immunology, University Hospital Zurich, Zurich, Switzerland), respectively. RR4-7 was purchased from BD Pharmingen (San Diego, CA, USA). Abs used for flow cytometry were recombinant anti NIP- or I-E^d ^hIgG_3 _described earlier [49,65], and PE-conjugated goat anti-hIgG F(ab)_2 _fragments from Southern Biotechnologies (Birmingham, AL).

### Generation of TCR-Ig fusion constructs

Cloning of TCR V genes and fusion to the C region of h IgG_3 _(TCRV-IgC in Figure 1A) has been described previously [57] as has cloning of complete TCR ectodomains (cTCR) and fusion to complete hIgG_3 _(cTCR-cIg in Figure 1B) [29]. In short, TCR α- and β-chain genes (V or V + C) were PCR amplified from 4B2A1 and 7A10B2 cDNA. PCR primers had restriction sites for cloning of TCR genes into the pLNOH2 and pLNOκ vectors [66]. The TCR V genes were introduced upstream from the Cγ3 C region in pLNOH2 and the C_κ _C region in pLNOκ, whereas the complete TCRs were cloned upstream from a complete hIgG_3 _heavy chain in pLNOH2 and a complete κ light chain in pLNOκ. For both formats and TCRs, TCRα was fused to either Ig heavy or light chain, as was TCRβ. In the case of the cTCR-cIg constructs, the primers were designed to introduce a segment encoding a GS-linker of six amino acids between the TCRs and the Ig.

For insect cell production, the pAc-κ-Fc vector from PROGEN Biotechnik (Heidelberg, Germany) was used. Initially, a BamHI site between the polyhedrin promoter and the heavy leader in the vector as well as an EcoRV site in the 4B2A1 β-chain were removed by *in vitro *mutagenesis. Primers are given in Additional file 1. TCR-Ig genes were PCR amplified from the pLNO vectors using primers tagged with restriction sites (Additional file 1) and subcloned into pAc-κ-Fc. For the TCRV-IgC constructs, the PCR products included the entire TCR-Ig fusions from pLNO vectors, and the inserts replaced the Fc region in the vector. XhoI and BamHI sites were used for the TCRV_α_-Ig heavy chains whereas SacI and EcoRV sites were used for the TCRV_β_-Ig light chains. For the cTCR-IgFc fusions (Figure 1C), the TCR α chains were amplified and introduced upstream from the IgG_1 _Fc region in the vector, whereas the TCR β chains were not fused to any Ig domain. The restriction sites used were XhoI and SpeI for the α-chain and SacI and EcoRV for the β-chain. All restriction enzymes were from New England Biolabs (Ipswich, MA).

### *In vitro *mutagenesis and selection of mutants

Introduction of a disulfide bridge between the murine TCR C domains and replacement of an unpaired β-chain cysteine, have been described previously [29]. All other *in vitro *mutagenesis reactions were performed using the QuikChange Site-Directed Mutagenesis Kit from Stratagene (La Jolla, CA). Mutagenesis with multiple sets of primers was performed using the same protocol as for single site mutations, except for the presence of more than one primer set in each tube. The mutations were S82R in 4B2A1 V_α_; G17E, H47Y, and L80S in 4B2A1 V_β_; L43P and W82R in 7A10B2 V_α_; and Q17E in 7A10B2 V_β_. The primers are listed in Additional file 2. The presence of multiple primer pairs reduced the frequency of mutation at each targeted site to approximately 50%. Following mutagenesis reactions and transformation of *E. coli*, cultures of single colonies were grown and stored. Six to eight clones from each reaction were sequenced, and all mutations were detected, and in different combinations in individual clones. A total of 129 (24 for 4B2A1 V_α_, 30 for 4B2A1 V_β_, 45 for 7A10B2 V_α_, and 30 for 7A10B2 V_β_) colonies were picked and screened.

For selection, up to five culture aliquots were combined in pools, from which plasmid DNA were isolated. HEK 293E cells were then transiently transfected (2.4) with these plasmid preparations such that all possible combinations of pooled DNA for the α- and β-chains were tested. Protein production after each transfection was measured using the hIgG-specific ELISA (2.7), and wells with increased TCRV-IgC production identified. Plasmid DNA preparations were then made from the cultures initially pooled, and these individual DNA preparations again combined in a new HEK 293E cell transfection to finally detect the best pair.

### Transfection of HEK 293E cells

HEK 293E cells were transfected using Lipofectamine 2000 transfection reagent from Invitrogen (Carlsbad, CA) essentially as described [57]. For small scale testing, cells were seeded in 24 well plates at 2 × 10^5 ^cells/well, and supernatants were tested three days later. For larger scale protein production, cells were seeded in 750 ml bottles at 2 × 10^7 ^cells/bottle. In these, medium was harvested and replaced with fresh medium every 2-3 days for three weeks.

### Baculovirus production and infection of insect cells

Generation of recombinant baculovira and infection of Sf9 insect cells was performed using the BaculoGold Transfection Kit from BD Biosciences (San Diego, CA). Briefly, samples of pAc vectors with TCR-Ig genes and baculovirus DNA were co-transfected into Sf9 cells to generate recombinant baculovira carrying TCR-Ig genes. After the initial transfection, virus titers were increased by three repeated Sf9 cell infections.

### Protein purification

TCR-Ig fusion proteins were purified from transfected HEK 293E cell supernatants. Dead cells were removed by centrifugation, and the supernatant filtered through a 0.45 μm filter and run on a Protein G Sepharose 4 Fast Flow column from GE Healthcare (Uppsala, Sweden). Bound protein was eluted with Tris-HCl, pH 2.7, and the pH in each 1 ml fraction rapidly neutralized with 40 μl Tris-HCl pH 9. Fractions with TCR-Ig (as determined in ELISA) were concentrated on Amicon Centrifugal Filter Devices with MWCO 100 000 from Millipore (Billerica, MA). After a 40-70-fold concentration, four rounds of PBS were added to and spun through the filter column to replace the elution buffer.

### Quantification of TCR-Ig fusion protein

A hIgG_3_-specific ELISA used to quantify TCR-Ig fusion protein has been described previously [57]. Briefly, wells were coated with mouse anti-hIgG_3 _(clone HP6047), and TCR-Ig fusion protein added. Biotinylated mouse anti-hIgG_3 _(clone HP6050) followed by streptavidin-coupled alkaline phosphatase (ALP) was used for detection. TCR-Ig fusion proteins on hIgG_1 _Fc were quantified with goat anti-hIgG Fc (1:1000) as coat and ALP-conjugated goat anti-hIgG (1:2000) as detection reagent.

### TCR-Ig binding to TCR specific antibodies

For TCR-specific ELISAs, wells were coated with 3 μg/ml anti-TCR Ab (GB113, F23.2, 44-22-1, RR4-7 or H57) before addition of TCR-Ig fusion proteins. Detection was with ALP-conjugated goat anti-hIgG (1:5000). All ELISAs were developed using phosphatase substrate dissolved in diethanolamine buffer, pH 9.8. Streptavidin-ALP was from Amersham Biosciences (Uppsala, Sweden) whereas phosphatase substrate tablets were from Sigma-Aldrich.

### Flow cytometry

A20 and F9 cells were incubated ON with synthetic λ2^315 ^peptide (amino acid 89-107) from Biopeptide Co (San Diego, CA) at a final concentration of 50 μM. Then, additional peptide was added to a final concentration of 100 μM. After two hours, the cells were stained with TCR-Ig (at 17 or 100 μg/ml) or control Abs, followed by PE conjugated goat anti-hIgG F(ab)_2 _at 7.5 μg/ml. Recombinant I-E- and NIP-specific hIgG_3 _were positive and negative isotype-matched controls, respectively. 30-50 000 cells were run on a FACSCalibur flow cytometer from Becton Dickinson (Mountain View, CA) and analyzed.

## Authors' contributions

EL participated in the study design, performed all the experimental work and drafted the manuscript. GÅL participated in the SS bridge design, result interpretation and helped drafting the manuscript. BB participated in the study design and provided key reagents. IS conceived the project, organized funding, supervised the study and finalized the manuscript. All authors read and approved the final manuscript.

## Supplementary Material

Additional file 1 **Primers for cloning into pAc-κ-Fc**. Click here for file

Additional file 2 **Primers for V region mutagenesis**. Click here for file
