# Supplementary material for: Stabilizing mutations increase secretion of functional soluble TCR-Ig fusion proteins
Source: BMC Biotechnol. 2010 Aug 24;10:61. doi: 10.1186/1472-6750-10-61 (PMC2936418; doi:10.1186/1472-6750-10-61)
Supplement: Additional file 1 — Primers for cloning into pAc-κ-Fc. [file 1472-6750-10-61-S1.DOC]

**Additional file 1.** Primers for cloning into pAc-κ-Fc

| **Primer name** | **Primer sequences*** |
| --- | --- |
| Δ BamHI forward | cctataaatacggatc**g**accatggagtttgggc |
| Δ BamHI reverse | gcccaaactccatggt**c**gatccgtatttatagg |
| Δ EcoRV forward | gcagcactgagaaaggaga**c**atccctgatggatacaagg |
| Δ EcoRV reverse | ccttgtatccatcagggat**g**tctcctttctcagtgctgc |
| **TCRV-IgC** |  |
| XhoI-4α forward | ctg*ctcgag*cagcagaaggtgcagc |
| XhoI-7α forward | ctg*ctcgag*cagtcagtgacacagccc |
| Cγ3-BamHI reverse | ggt*ggatcc*tcatttacccggagacag |
| SacI-4β forward | att*gagctc*gaggctgcagtcaccc |
| SacI-7β forward | att*gagctc*ggtggcatcattactcagac |
| Cκ-EcoRV reverse | aga*gatatc*ctaacactctcccctgttgaagctctttgtgacgggc**c**agctcaggccct |
| **cTCR-IgFc** |  |
| XhoI-4α forward | ctg*ctcgag*cagcagaaggtgcagc |
| 4α-SpeI reverse | aca*actagt*ttgaaagtttaggttcatatctgtttc |
| SacI-4β forward | att*gagctc*gaggctgcagtcaccc |
| 4β-EcoRV reverse | aga*gatatc*tcagatctcatagaggatggtg |
| * Bold nucleotides are mutated; Enzyme sites are in italics; Mutation that removes SacI site in Cκ is underlined.  | |
