# Supplementary material for: Stabilizing mutations increase secretion of functional soluble TCR-Ig fusion proteins
Source: BMC Biotechnol. 2010 Aug 24;10:61. doi: 10.1186/1472-6750-10-61 (PMC2936418; doi:10.1186/1472-6750-10-61)
Supplement: Additional file 2 — Primers for V region mutagenesis. [file 1472-6750-10-61-S2.DOC]

**Additional file 2.** Primers for V region mutagenesis

| **Mutation** | **Primer Sequences*** |
| --- | --- |
| **4B2A1 Vα** |  |
| S82R forward | ctcccagcccag**a**gactctgctctc |
| S82R reverse | gagagcagagtc**t**ctgggctgggag |
| **4B2A1 Vβ** |  |
| G17E forward | gcagtaacaggag**a**aaaggtgacattg |
| G17E reverse | caatgtcaccttt**t**ctcctgttactgc |
| H47Y forward | gctgaggctgatc**t**attattcatatgg |
| H47Y reverse | ccatatgaataat**a**gatcagcctcagc |
| L80S forward | cattctggagt**c**ggctaccccctc |
| L80S reverse | gagggggtagcc**g**actccagaatg |
| **7A10B2 Vα** |  |
| L43P forward | gcggcaggggc**c**gcagctgctcc |
| L43P reverse | ggagcagctgc**g**gcccctgccgc |
| W82R forward | cctccgtgcac**c**ggagcgactcg |
| W82R reverse | cgagtcgctcc**g**gtgcacggagg |
| **7A10B2 Vβ** |  |
| Q17E forward | gtcaggaaggg**g**aaaaactgacc |
| Q17E reverse | ggtcagttttt**c**cccttcctgac |
| * Bold nucleotides are mutated | |
